# COVID-19 Vaccination in Patients with Classic Kaposi’s Sarcoma

**DOI:** 10.3390/vaccines9060632

**Published:** 2021-06-10

**Authors:** Alice Indini, Athanasia Tourlaki, Francesco Grossi, Donatella Gambini, Lucia Brambilla

**Affiliations:** 1 Medical Oncology Unit, Fondazione IRCCS Ca’ Granda, Ospedale Maggiore Policlinico, 20122 Milan, Italy; donatella.gambini@policlinico.mi.it; 2 Dermatology Unit, Fondazione IRCCS Ca’ Granda, Ospedale Maggiore Policlinico, 20122 Milan, Italy; athanasia.tourlaki@policlinico.mi.it (A.T.); lucia.brambilla@policlinico.mi.it (L.B.); 3 Medical Oncology Unit, Department of Medicine and Surgery, University of Insubria, ASST dei Sette Laghi, 21100 Varese, Italy; francesco.grossi@asst-settelaghi.it

**Keywords:** COVID-19, vaccination, Kaposi’s sarcoma, SARS-CoV-2, cancer, HHV-8 DNA

## Abstract

The novel coronavirus disease 2019 (COVID-19) has represented an overwhelming challenge for worldwide health systems. Patients with cancer are considered at higher risk for severe COVID-19 and increased mortality in case of infection. Although data on the novel severe acute respiratory syndrome coronavirus 2 (SARS-CoV-2) vaccination in patients with cancer are limited, there is enough evidence supporting anti-infective vaccination in general in patients with active cancer, or with history of previous malignancy. Subjects with classic Kaposi’s sarcoma (KS) represent a small subset of cancer patients, which should be considered at heightened risk for infections due to several factors including age, and impaired immune function status. Several cases of human herpesviruses reactivation among critically ill COVID-19 patients have been described. Moreover, in case of severe infection and treatment with immunomodulating agents, patients with CKS are exposed at significant risk of viral reactivation and disease progression. Considering the baseline clinical risk factors of patients with CKS, and the complex interplay of the two viral agents, SARS-CoV-2 vaccination should be strongly recommended among patients with KS. KS represents an interesting field to study the interactions among chronic viral infections, SARS-CoV-2 and the host’s immune system. Prospective observational studies are needed to provide more insights on vaccine activity and safety among patients with cancer, optimal vaccine schedules, potential interactions with antineoplastic therapies, and other comorbidities including chronic viral infections.

The novel coronavirus disease 2019 (COVID-19) has represented an overwhelming challenge for worldwide health systems [1]. Since the onset of the COVID-19 pandemic, there has been growing concern regarding subjects with chronic disease and poor health status, including patients with cancer [2]. Immunosuppression, chronic inflammation, and concomitant myelosuppressive therapies have been listed as major causes of higher risks of complications related to COVID-19 among cancer patients [3,4]. These observations were supported by early evidence of poorer survival outcomes of COVID-19 in patients with cancer as compared with subjects with no history of malignancy, even though most observations were based on non-comparative retrospective studies [2,5,6]. COVID-19 may also result in significant diagnostic and treatment delays in patients with cancer, with potential negative impact on cancer-related morbidity and mortality. Oncology practices have been significantly reorganized, in order to minimize the risk of severe acute respiratory syndrome coronavirus 2 (SARS-CoV-2) exposure and COVID-19 spread among cancer patients, along with maintaining timely diagnosis and treatment in oncology facilities [7].

Recently, considerable efforts have been put into developing safe and effective vaccines against SARS-CoV-2 [8]. The main techniques of SARS-CoV-2 vaccine development relied on technologies based on messenger RNA (mRNA), inactivated/attenuated or genetically modified viruses, synthetic long viral peptides and plasmid DNA vaccines [8]. To date, several vaccines have gained the European Medicines Agency (EMA) approval, and many others are currently being tested for efficacy and safety in placebo-controlled phase III studies. Overall, mRNA-based vaccines have shown a 90–95% protection from COVID-19, whereas non-replicating adenoviral vector-based vaccines have shown protection rates of 70–90% [9,10]. Both types of vaccines promote the intracellular production of SARS-CoV-2 spike protein, stimulate the production of type I interferon (IFN), leading to the differentiation of CD4+ and CD8+ T cells, which sustain inflammatory cytokines production, and CD4+ T helper, which promote B cell differentiation and antibodies production [11].

Although data on SARS-CoV-2 vaccination in patients with cancer are extremely limited [12], there is enough evidence to support anti-infective vaccination in general in patients with active cancer, or history of previous malignancy. In this subpopulation of patients, the use of live-attenuated vaccines and replication-competent vector vaccines should usually be avoided [13,14]. In fact, unlike inactivated, or nucleic acid vaccines, live-attenuated vaccines may pose a risk of replication of the virus after administration, while live vaccines can actually induce infection in immunocompromised patients [15], implying a careful evaluation of the vaccines’ risk–benefit profile. Lessons learned from other vaccinations (e.g., influenza, pneumococcus, hepatitis B) suggest that immunization in patients with cancer significantly reduces the risk of vaccine-preventable infections and decreases the number of vulnerable patients able to disseminate the pathogen [16].

Several guidelines have been promptly implemented in order to include indications on COVID-19 vaccination in patients with cancer [17]. At the present time, patients with active cancer and those receiving anti-cancer treatment, have been prioritized for immunization with any of the two available mRNA-based vaccines (i.e., Pfizer/BioNTech [BNT162b2 mRNA vaccine], and Moderna [mRNA-1273 SARS-CoV-2 Vaccine]). In fact, from a safety standpoint, results of the main mRNA vaccines’ trials have not demonstrated vaccine-related serious adverse events, and have provided the highest protection rates [9].

However, there might be significant differences for severe COVID-19 among cancer patients regarding underlying tumor (i.e., primary tumor site, stage of disease), and type of systemic treatment (i.e., chemotherapy, immunotherapy, targeted therapy) [18,19]. Specific clinical factors (e.g., age, hematological malignancy, recent chemotherapy), and anti-cancer therapies (e.g., R-CHOP, platinum combined with etoposide, and DNA methyltransferase inhibitors) have been associated with poorer outcomes among patients with cancer and COVID-19 [20].

Subjects with classic Kaposi’s sarcoma (CKS) represent a small subset of cancer patients, which should be considered at heightened risk for infections due to several factors including age, and impaired immune function status [18]. Kaposi’s sarcoma (KS) is a chronic infection caused by the human herpes virus-8 (HHV-8), also known as KS-associated herpesvirus (KSHV) [21]. This oncogenic virus has two alternating life-cycle programs, the latent and the lytic phases, which both play a pivotal role in the initiation and progression of KSHV-induced cancers (KS, primary effusion lymphoma, multicentre Castleman’s disease and plasmablastic lymphoma) [22,23]. The pathogenesis of KS also relies on an immunodeficiency status, and recent evidences have underlined the potential role of immune checkpoint inhibitors in this rare disease, exploiting the immunogenicity of virus-associated antigen [24,25]. Several cases of human herpesviruses reactivation among critically ill COVID-19 patients have been described [26,27], together with an increased number of Herpes Zoster cases during the COVID-19 pandemic [28,29]. It has also been reported that SARS-CoV-2 encoded proteins and some anti-COVID-19 drugs can induce lytic reactivation of KSHV in vitro, thereby promoting initiation of oncogenesis and viral dissemination [30]. Moreover, both KSHV and SARS-CoV-2 strongly reduce type I IFN production, which is already in itself impaired in older patients due to immunosenescence, with potential detrimental effects in case of coinfection [31,32].

Overall, these evidences suggest that patients with CKS may be at high risk of COVID-19. Moreover, in case of severe infection and treatment with immunomodulating drugs (e.g., corticosteroids in combination with anti-interleukin 6 receptor antibody, tocilizumab, or with the Janus kinase (JAK) 1/2 inhibitor, baricitinib) [33], patients with CKS are exposed at significant risk of viral reactivation and disease progression. However, CKS patients aged <80 years and those who are not receiving active chemotherapy have not been considered a high-priority category of cancer patients for COVID-19 vaccination from the very beginning of vaccination programs. Considering the baseline clinical risk factors of patients with CKS, and the complex interplay of the two viral agents, in our opinion, SARS-CoV-2 vaccination should be strongly recommended among CKS patients. Previous evidence has supported the immunogenicity and safety of influenza vaccination in this subset of patients [34]. We therefore propose regular follow up of patients with CKS during the present COVID-19 pandemic, and global adhesion to the vaccination program, with periodic clinical, and possibly with serological monitoring of HHV8 DNA and antibodies before and after vaccine administration. Considerations on patients with CKS are far from being generalized to include all other patients with cancer, due to the peculiarity of CKS pathogenesis, the chronic evolution, and the rarity of the disease. However, CKS represents an intriguing field to study the interactions among chronic viral infections, SARS-CoV-2 and the host’s immune system. Observational studies should be pursued to provide more insights on vaccine activity and safety among patients with cancer, optimal vaccine schedules, potential interactions with antineoplastic therapies, and other comorbidities including chronic viral infections. Prospective data collection in patients with CKS is currently undergoing at our Institution, in order to clarify the mechanisms of SARS-CoV-2 interaction with cancer cells and the immune system, with potential positive implications in future research in this field.
